# Arrayed Micropillar Ionic Film Iontronic Flexible Pressure Sensor and Its Wearable Sensing Applications

**DOI:** 10.3390/mi17090995

**Published:** 2026-08-23

**Authors:** Wenzhen Liang, Xiaodong Huang

**Affiliations:** 1 School of Automation and Information Technology, Guangdong Polytechnic of Water Resources and Electric Engineering, Guangzhou 510635, China; 2 Department of Intelligent and Information Engineering, Taiyuan University, Taiyuan 030051, China; 2024502001@tyu.edu.cn

**Keywords:** iontronic flexible pressure sensor, ionic film, array micro-pillars, TPU elastic matrix, electronic skin

## Abstract

Flexible pressure sensors serve as core sensing components for wearable health monitoring systems, electronic skins for soft robots, and flexible human–machine interaction devices. Benefiting from the interfacial electric double-layer polarization effect, iontronic sensing delivers far higher pressure response sensitivity than conventional parallel-plate capacitive sensors, endowing it with distinctive advantages in the detection of weak physiological signals. Nevertheless, current dense ionic thin-film dielectric layers suffer from limited deformation space under compression and poor low-pressure sensing capability. Mainstream high-precision micropillar arrays are fabricated via photolithography, 3D printing, and metal etching molds, which require costly equipment and complicated fabrication procedures, making large-area mass production unfeasible. Random frosted concave-convex microstructures feature disordered dimensions, leading to severe device hysteresis and narrow linear ranges, which fail to achieve ultrahigh sensitivity alongside a wide pressure detection range simultaneously. To address the aforementioned multiple bottlenecks, this paper proposes a low-cost resin template replication process to fabricate TPU-based ionic thin-film dielectric layers with ordered micropillar array microstructures. Combined with inkjet-printed silver conductive PI flexible electrodes, an iontronic flexible pressure sensor with a sandwich layered structure is constructed. Multi-dimensional investigations including microscopic morphology characterization, electromechanical sensing performance calibration, and human wearable application tests are systematically implemented to thoroughly elucidate the synergistic enhancement mechanism of the arrayed micropillars. Test results demonstrate that the effective pressure detection range of the sensor spans 0–1038 kPa, accommodating ultra-low pressures such as pulse signals as well as medium-to-high-pressure loads including joint bending. The sensitivity reaches 23.27 kPa^−1^ within the low-pressure range of 0–200 kPa and remains stable at 3.52 kPa^−1^ in the high-pressure range of 200–1038 kPa, with piecewise linear fitting correlation coefficients of 0.93 and 0.96 respectively. Both the response time and recovery time of the device are 40 ms, and the hysteresis error throughout the loading-unloading cycle is merely 2.62%. After 20,000 consecutive cyclic loading-unloading tests, the peak capacitance output only decays by 5.1%, verifying outstanding mechanical fatigue resistance and electrical stability. Validations in multi-scenario applications prove that the sensor can accurately capture human physiological and motion signals including radial artery pulses, laryngeal deformation induced by multi-syllable vocalization, and multi-angle bending of fingers and elbow joints, suitable for home-based health monitoring, quantitative rehabilitation training, flexible tactile interaction and other scenarios. The entire fabrication process eliminates high-precision micro-nano processing equipment such as photolithography systems, plasma etchers and 3D printers; only general chemical raw materials and conventional laboratory instruments are adopted. The reusable templates enable low manufacturing costs and large-area coating forming, offering a novel low-cost technical solution for the engineering implementation and industrialization of high-performance iontronic flexible pressure sensors.

## 1. Introduction

The technologies of new-generation artificial intelligence, flexible electronics, smart healthcare and soft robots are undergoing rapid iterations. Demands such as real-time human body surface pressure sensing, tactile recognition of curved objects and continuous monitoring of weak physiological signals keep surging, making flexible pressure sensing technology a core research direction spanning the interdisciplinary fields of materials, measurement and control, and biomedicine [1,2,3,4]. Conventional metal strain gauges and ceramic piezoresistive elements feature high rigidity and extremely poor ductility. They cannot fit the irregular curved surfaces of human bodies and special-shaped workpieces, tend to cause foreign body sensation after long-term wear, and fail to support thin-film and lightweight design, which severely restricts their applicable scenarios [5,6,7]. Flexible pressure sensors fabricated from polymer elastic materials including thermoplastic polyurethane (TPU) and polydimethylsiloxane (PDMS) possess prominent advantages such as light weight, outstanding bendability, favorable biocompatibility and convenient thin-film processing. They have gradually replaced rigid sensing components and become the mainstream research and development focus at present [8,9,10].

Classified by the physical mechanism of force-electric conversion, existing flexible pressure sensors are mainly divided into the following four major systems: piezoresistive [11], piezoelectric [12], triboelectric nanogenerator-based [13], and conventional capacitive [14]. Devices of each type show remarkable differences in material systems, measuring range adaptability, power consumption, stability, and fabrication cost. Among them, capacitive sensors have attracted extensive research attention owing to their simple structure, excellent static stability, and ultra-low operating power consumption [15,16]. However, conventional capacitive sensors adjust capacitance merely via bulk polarization of dielectrics, resulting in limited variation in electrical signals under compression. Their sensitivity in the tiny pressure range cannot be significantly improved, which acts as a critical bottleneck restricting their widespread application in high-precision wearable electronics. The iontronic sensing mechanism was formally proposed by Pan’s research group [17] in 2011. Its core principle lies in the formation of a Helmholtz electric double layer at the interface between electrodes and ionic thin films. Slight interfacial deformation can drive the directional migration of anions and cations, yielding capacitance changes increased by orders of magnitude. This perfectly compensates for the insufficient low-pressure sensitivity of traditional capacitive sensors, making iontronic sensors a key research focus in the global flexible sensing field over the past decade [18,19,20]. As the core sensitive medium of iontronic sensors, ionic thin films are fabricated by blending and curing polymer elastic matrices with room-temperature ionic liquids. Free anions and cations inside the films can rapidly accumulate at electrode interfaces under the combined effect of external electric fields and mechanical forces, serving as the pivotal material for ultra-high-sensitivity pressure detection. Currently, there are two primary approaches to optimize the performance of ionic thin film devices as follows: the first is modification of polymer matrices, where copolymerization and nanofiller doping are adopted to enhance material elasticity and electrical conductivity; the second is constructing microstructures on the surface or inside dielectric layers to reduce elastic modulus and boost deformability under compression. Numerous studies have verified that microstructure regulation represents the most efficient and straightforward technical strategy to enhance the sensitivity of iontronic sensors. The mainstream microstructures fall into the following two categories: surface protrusion arrays (micropillars, micropyramids, and microdomes) and internal three-dimensional porous networks [21,22,23].

In recent years, microstructure modification strategies have been widely adopted to enhance the sensing performance of ionic liquid-based sensors. Nevertheless, current approaches still suffer from multiple irreconcilable technical conflicts. First, the fabrication cost of ordered micropillar arrays remains excessively high. At present, high-precision ordered micropillars are mostly manufactured via ultraviolet photolithography, metal-etched master molds, and photocurable 3D printing, which incur expensive equipment procurement and maintenance fees. Custom molds require a fabrication cycle of several days and can only produce small-batch laboratory specimens, failing to meet the demands of large-area mass production. Wan et al. [24] fabricated capacitive sensors with micro-tower structures using lotus leaf biomimetic templates. Although the fabrication process was simplified, the natural biomimetic templates exhibited poor dimensional uniformity, leading to significant performance dispersion across device batches. Yang et al. [25] manufactured micropillar electrodes through photolithography and wet etching, achieving a low-pressure sensitivity of 7.68 kPa^−1^ within the range of 0–0.4 kPa, yet the effective detection range was limited to merely 4 kPa, which cannot accommodate medium and high-pressure scenarios such as human joint movement and mechanical clamping. Second, low-cost random microstructures possess prominent performance drawbacks. Concave-convex random microstructures replicated from frosted glass and sandpaper eliminate the need for precision equipment and come at a low cost. However, their randomly distributed microscopic protrusions cause localized stress concentration under compression, readily triggering irreversible plastic deformation. This results in substantial hysteresis error, poor cycling stability, a drastic sensitivity gap between low and high-pressure ranges, and low linear fitting goodness [26,27,28]. Third, existing devices lack balanced comprehensive performance. Certain ultra-high-sensitivity sensors are only applicable to ultra-low pressure at the hectopascal level with an extremely narrow detection range; devices with wide measurement ranges generally deliver weak low-pressure sensing capacity, making it difficult to simultaneously satisfy the dual detection requirements of weak physiological signals and industrial mechanical pressure. Furthermore, most relevant studies only conducted cycling tests within one thousand cycles, lacking data support for long-term stable wearable operation [29,30,31].

To balance sensing performance, fabrication cost and mass production adaptability, this study uses highly elastic TPU1185A as the matrix and blends it with [EMIM]+[TFSI]-imidazolium ionic liquid to fabricate ionic sensing films. A recyclable resin template is adopted for spin-coating and reverse molding to prepare ordered micro-pillar dielectric layers, which are combined with inkjet-printed silver flexible PI electrodes to assemble sandwich-structured ionic flexible pressure sensors. Innovations are achieved in the following four dimensions: process, structure, mechanism and application. Micro-pillars are molded with resin templates without high-cost equipment such as photolithography and 3D printing. The raw materials are commonly available and the large-area fabrication is achievable, which effectively cuts mass production costs. The regular micro-pillar array features the following hierarchical compression behavior: the interstitial air amplifies low-pressure signals while the TPU matrix stabilizes high-pressure output, balancing high and low pressure sensitivity and enabling wide-range detection from 0 to 1038 kPa. A coupled sensing model combining geometric capacitance and electric double-layer capacitance is established to elaborate the sensitivity enhancement mechanism, wherein micro-pillars modulate the electrode plate structure and accelerate ion migration. The practicality of the device is verified via physiological signal tests on multiple human body parts, broadening its application scenarios in wearable medical treatment and human–machine interaction. This paper completes a full set of experiments including material preparation, device fabrication, morphological characterization, electrical calibration and practical scenario testing. The core performances of devices with different dielectric layers are compared, providing a complete experimental protocol and theoretical support for the research and development of low-cost, high-performance ionic flexible pressure sensors.

## 2. Experimental Section

### 2.1. Standard Test Environment for Experiments

Unified standard test environment: ambient temperature of 25 ± 2 °C and ambient relative humidity of 40%RH to avoid interference of temperature and humidity fluctuations on ion migration and deformation of elastic materials; the loading rate of the press is fixed at 0.5 mm/min for quasi-static pressurization to eliminate signal errors caused by dynamic impact loads; 5 parallel samples are prepared for each formula, and each performance test is repeated 3 times to take the average value so as to eliminate discrete preparation errors. Impedance Analyzer (Keysight E4990A, Keysight Technologies, Santa Rosa, CA, USA), measurement frequency: 1 kHz, AC excitation amplitude: 100 mV, DC bias: 0 V, parallel capacitance test mode, sampling frequency: 10 Hz, built-in first-order low-pass filter (cut-off frequency: 5 Hz); the no-load initial capacitance C_0_ is uniformly set to 25 ± 2 pF; pressure loading equipment (INSTRON 5567 universal testing machine, Instron Corporation, Norwood, MA, USA), force sensor accuracy: ±0.01 N, compression platen area: 1 cm^2^, effective sensing area of the device: 10 mm × 10 mm; pressure conversion formula: P = F/S; preloading with 0.5 N pre-pressure, holding pressure for 5 s at each pressure level, loading/unloading rate fixed at 0.5 mm/min; morphological characterization: field emission scanning electron microscope (SU8010, Hitachi High-Technologies Corporation, Tokyo, Japan) with an acceleration voltage of 5 kV; and Dektak XT profilometer (Bruker Nano Surfaces Division, Tucson, AZ, USA) adopted to measure the heights of thin films and micro-pillars.

### 2.2. Preparation of Resin Square Microcolumn Master Mold

No photolithography or etching is adopted throughout the fabrication of the master mold. The processing procedure is as follows: numerical control milling machines are used to mechanically mill epoxy resin plates to directly fabricate periodic square groove arrays. The grooves feature a side length of 400 μm, a depth of 160 μm, and a center-to-center spacing of 800 μm within the array. The minimum machining precision of the milling cutter is ±0.5 μm. The entire process relies solely on mechanical cutting without employing any photoresist, mask, or plasma etching techniques. After milling, the inner walls of the grooves are polished, ultrasonic cleaning is performed to remove cutting debris, and drying is carried out at 100 °C for 2 h to obtain the replicated resin master mold. Continuous spin-coating is conducted to replicate thin films using a single master mold. The dimensional error of micropillars remains steadily ≤7% for the first 80 replicated samples. Slight wear occurs at the groove edges after 85 replication cycles, pushing the dimensional error of micropillars up to 12%. Accordingly, the effective reusable times of the master mold are no less than 80 replications.

### 2.3. Preparation Process of Dielectric Layer with Arrayed Microcolumn Ion Membrane

**Figure 1:**
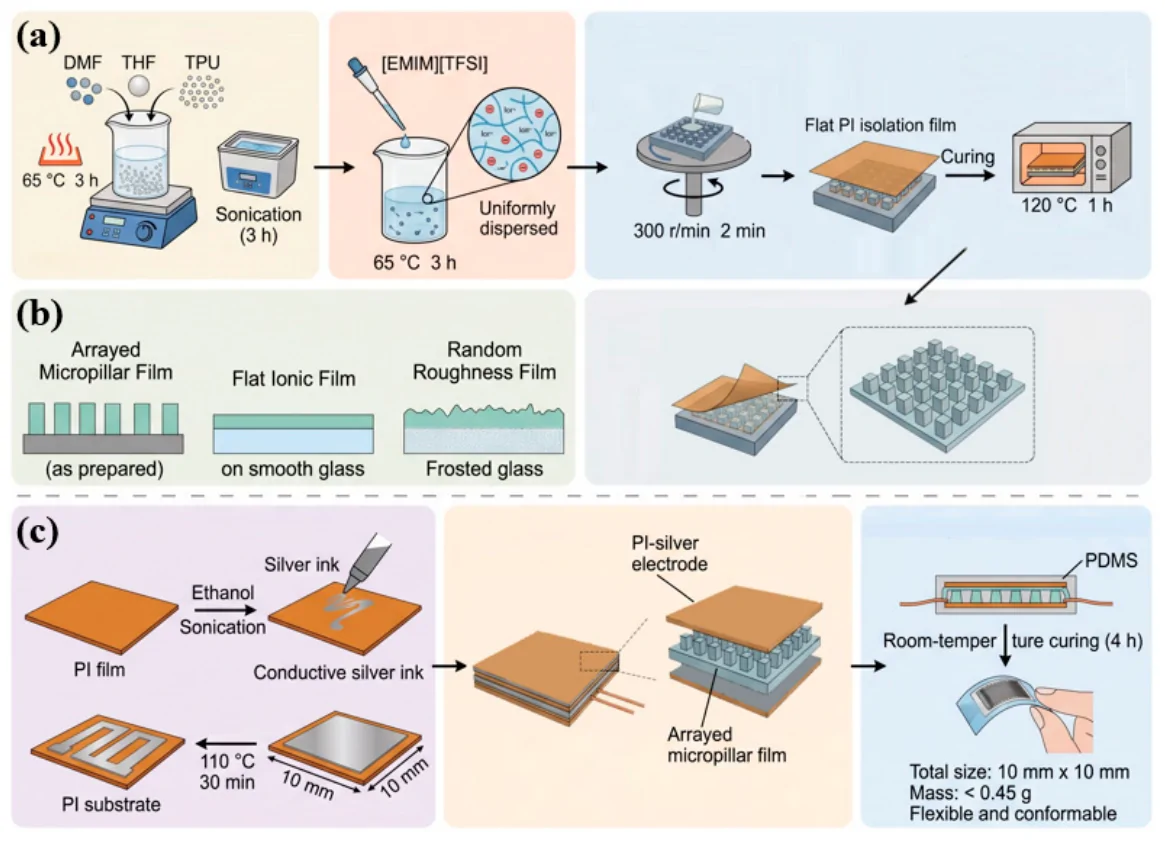
Sensor preparation process: (**a**) fabrication process of the dielectric layer for sensors, (**b**) fabrication of the dielectric layer for control samples and (**c**) preparation of flexible conductive electrodes and packaging and assembly of devices.

The fabrication process of the sensor is illustrated in Figure 1. First, the TPU base solution is prepared: 20 mL of DMF and 20 mL of THF organic solvents are measured and mixed uniformly. Then, 4 g of TPU pellets subjected to drying pretreatment are added into the mixed solvent, followed by magnetic stirring at a constant temperature of 65 °C for 3 h to fully dissolve the TPU pellets and obtain a transparent, flocculate-free homogeneous solution. The resulting solution is then transferred to an ultrasonic cleaner for continuous sonication for 3 h to effectively break up polymer aggregates, yielding a TPU premixed base liquid with outstanding stability. After the preparation of the base liquid, 3 mL of [EMIM]+[TFSI]-ionic liquid with a purity of 99.5% is added to the system, and the mixture undergoes ultrasonic stirring at a low temperature of 65 °C for another 3 h. This step ensures the uniform dispersion of anions and cations within the TPU polymer matrix, eliminates local ion enrichment, and prevents discrepancies in interfacial impedance caused by uneven ion distribution. The prepared ionic composite solution is cast evenly onto the surface of a resin micropillar master mold and then spin-coated using a spin coater at a rotational speed of 300 r/min for 2 min. Centrifugal force drives the solution to fully fill the gaps between micropillars and form a smooth, uniform liquid film on the template surface. A flat PI separator film is subsequently laid over the top of the liquid film to isolate high-temperature airflow inside the oven and avoid cracking or damage of the thin film. The entire sample is placed in an electrothermal blast drying oven for constant-temperature curing at 120 °C for 1 h, allowing complete solvent evaporation and sufficient crosslinking and molding of TPU molecular chains. After the oven naturally cools down to room temperature, the resin master mold is peeled off slowly to acquire an ionic thin film with regularly arranged array micropillars. Multi-point thickness calibration of the sample is carried out via a step profiler, which reveals that the overall thickness of the thin film stabilizes at 100 μm, the protrusion height of micropillars is 26 μm, and the overall dimensional error of the micropillar array can be controlled within 7%. To conduct controlled experiments with a single variable and accurately analyze the influence of microstructures on sensing performance, two groups of control samples are fabricated simultaneously under identical raw material ratios, stirring procedures, curing temperatures and durations. The first control sample is a dense ionic thin film without microstructures formed by spin-coating and curing on a smooth glass substrate, while the second one is an ionic thin film with random concave-convex microstructures replicated from a commercial frosted glass template.

### 2.4. Preparation of Flexible Conductive Electrodes and Packaging and Assembly of Devices

1. Fabrication of Flexible Silver Electrodes: Wipe the PI film substrate with anhydrous ethanol and conduct ultrasonic cleaning for 15 min, followed by drying to remove surface oil stains. Use an inkjet printing device to uniformly print conductive silver ink on one side of the PI film; then, dry the film in an oven at 110 °C for 30 min to completely remove ink solvents and form a continuous conductive electrode layer with an effective sensing area of 10 mm × 10 mm.
2. Sandwich Structure Assembly: Place the array micropillar ionic film dielectric layer on the surface of the lower PI silver electrode with the protruding side of micropillars facing upward, and fully attach it to the upper silver electrode to ensure complete contact between micropillar tops and the electrode. Lead oxygen-free copper wires out from the edges of upper and lower electrodes to serve as capacitive signal acquisition channels.
3. Sealing and packaging treatment: A thin layer of mixed PDMS adhesive is coated around the periphery of the device, followed by standing at room temperature for 4 h for full curing. This prevents air, water vapor and dust from penetrating into the ionic film and improves the environmental stability of the device. After being placed in an environment with 80% high humidity for 24 h and immersed in artificial sweat for 12 h, the sensitivity attenuation of the device is no more than 3.2%. PDMS can effectively block water vapor permeation and restrain the precipitation and leakage of ionic liquid. After packaging, the overall dimensions of the device are 10 mm × 10 mm with a total weight less than 0.45 g. Free of hard rigid components, the device can be bent arbitrarily and fitted closely to human body curved surfaces.

### 2.5. Calculation Methods of Electrical Properties

Sensor sensitivity serves as the core evaluation indicator, and its calculation formula is defined as follows:

(1) $$S=\frac{\Delta C/C_{0}}{\Delta P}=\frac{(C-C_{0})/C_{0}}{\Delta P}$$

In the formula: *S* stands for the sensor sensitivity with the unit of kPa^−1^; *C*_0_ refers to the initial capacitance of the device under no-load and zero pressure; C denotes the real-time capacitance value after pressure application; Δ*C* = *C* − *C*_0_ represents the capacitance variation; and ΔP is the pressure variation with the unit of kPa.

## 3. Working Principle of Devices and Characterization Analysis of Micro-Morphology

### 3.1. Dual-Capacitor Cooperative Coupling Sensing Mechanism Iontronic Sensors

The total equivalent capacitance of the arrayed micropillar iontronic pressure sensor in this paper is formed by the superposition and coupling of two components, namely the parallel-plate geometric capacitance *C_geo_* and the electric double-layer capacitance *C_EDL_* at the electrode-ion film interface. The total capacitance satisfies the relational expression:

(2) $$C_{total}=C_{geo}+C_{EDL}$$

The capacitances of the two types of capacitors increase positively synchronously with external pressure, generating a synergistic amplification effect. This is the core reason why the ionic sensor boasts far higher sensitivity than conventional purely geometric capacitor devices.

Fundamental formula for parallel-plate geometric capacitance:

(3) $$C_{geo}=\frac{\varepsilon_{0}\varepsilon_{r}A}{d}$$

**Figure 2:**
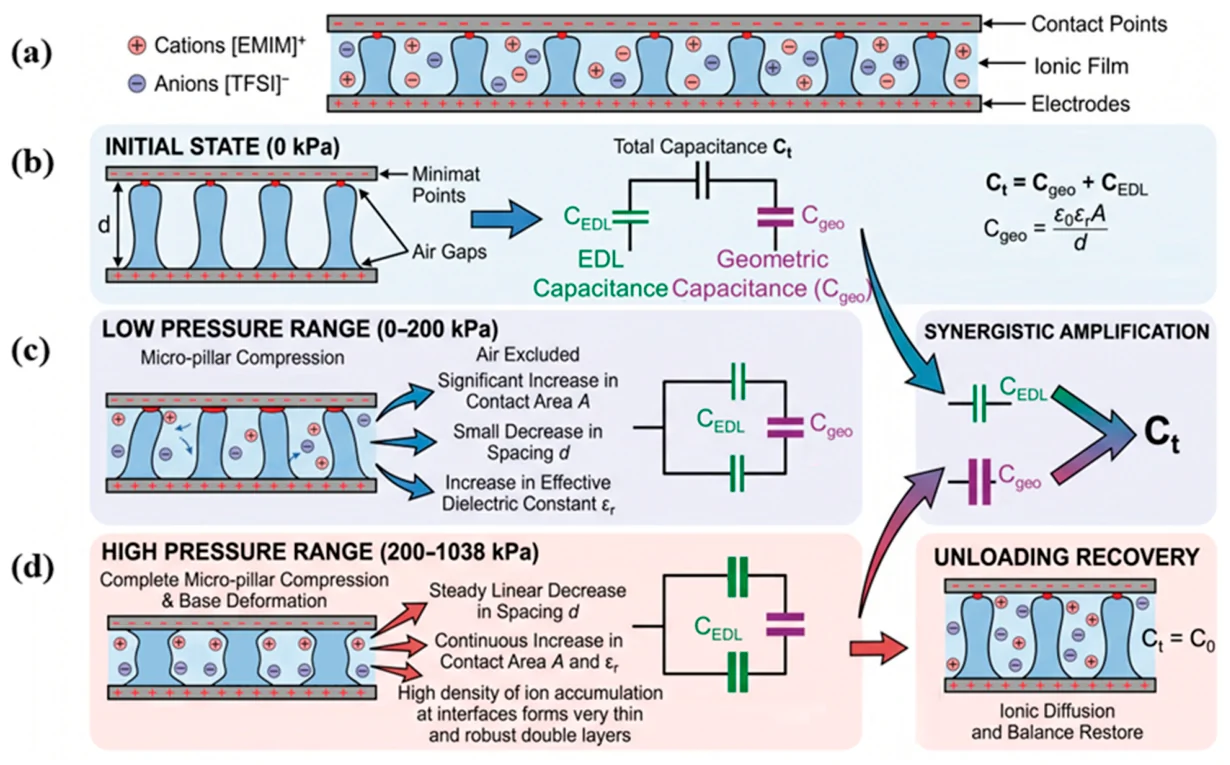
Working principle of the sensor: (**a**) schematic diagram of the sensor structure, (**b**) schematic diagram of the sensor under initial zero pressure state, (**c**) schematic diagram of the sensor under low pressure condition and (**d**) schematic diagram of the sensor under high-pressure condition.

ε_0_ stands for the permittivity of free space (8.854 × 10^−12^ F/m); ε_r_ represents the equivalent relative permittivity of the ionic thin film; *A* denotes the effective contact area between the electrodes and the dielectric layer; and d refers to the distance between the upper and lower electrode plates, as shown in Figure 2a.

As shown in Figure 2b, in the initial state with no external pressure applied, the gaps between the arrayed micropillars are completely filled with air (the relative permittivity of air ≈ 1), resulting in a low overall equivalent relative permittivity εr of the ionic film. Only a small number of points at the top of each micropillar are in contact with the upper electrode, which leads to a small effective contact area A and the maximum electrode plate spacing d, so the geometric capacitance C_geo_ remains at an extremely low level. Meanwhile, the [EMIM]^+^ cations and [TFSI]^−^ anions inside the ionic film are uniformly and randomly distributed without directional migration. The electric double layer at the interface between the electrodes and the film is in a thermodynamic equilibrium state with a large electric double layer thickness and a stable C_EDL_ value, and the device maintains a constant initial capacitance C_0_.

When vertical pressure is applied to the upper surface of the device, the arrayed micropillar structure undergoes hierarchical elastic compression, altering geometric capacitance parameters in the following two distinct stages:

(1) Low-pressure range (0–200 kPa), as shown in Figure 2c: Under low mechanical load, only the tops of micropillars bend and collapse, and air trapped within the gaps between pillars is rapidly squeezed out. On the one hand, compression of micropillars drastically multiplies contact points between electrodes and the film, leading to a marked increase in effective contact area A. On the other hand, the electrode plate spacing d slightly decreases, the air phase fraction drops, the film becomes denser, and the relative permittivity εr rises simultaneously. The combined effect of these three factors drives a rapid surge in geometric capacitance Cgeo.
(2) High-pressure range (200–1038 kPa), as shown in Figure 2d: All micropillars are fully compacted with no room for independent deformation. The entire structure undergoes uniform elastic compression relying solely on the TPU film substrate. The electrode plate spacing d decreases linearly on a continuous basis while εr grows steadily. This enables stable and continuous variation in geometric capacitance with applied pressure and extends the effective detection range of the device.

The electric double-layer capacitance is simultaneously enhanced under pressure: micropillar compression generates more electrode-film contact interfaces. Driven by an applied alternating electric field, free anions and cations inside the film rapidly migrate directionally—cations accumulate at the negative electrode interface, and anions gather at the positive electrode interface. The quantity of bound charges on electrode surfaces rises sharply, and the effective thickness of the electric double layer is compressed. According to the Helmholtz electric double-layer model, both expanded interfacial contact area and reduced double-layer thickness contribute to a prominent increase in CEDL. The synchronous growth of geometric capacitance and electric double-layer capacitance creates superimposed and amplified dual signals, delivering ultra-high capacitive response under tiny pressures.

After complete removal of external load, the TPU polymer matrix exhibits outstanding resilience, and collapsed micropillars gradually revert to their original upright shape. The electrode plate spacing d, effective contact area A and relative permittivity εr all return to their initial states. Meanwhile, anions and cations accumulated at interfaces break free from electrostatic confinement and diffuse evenly back into the bulk film, restoring equilibrium of the electric double layer. The total capacitance reversibly falls back to the initial value C0, completing one full reversible force-electricity conversion cycle. The ordered array of micropillars evenly distributes applied force, significantly mitigating overall film creep and hysteresis of ion migration, and effectively reducing loading-unloading hysteresis error of the device.

### 3.2. Characterization Results of Microscopic Morphology of Arrayed Micro-Pillar Dielectric Layers

**Figure 3:**
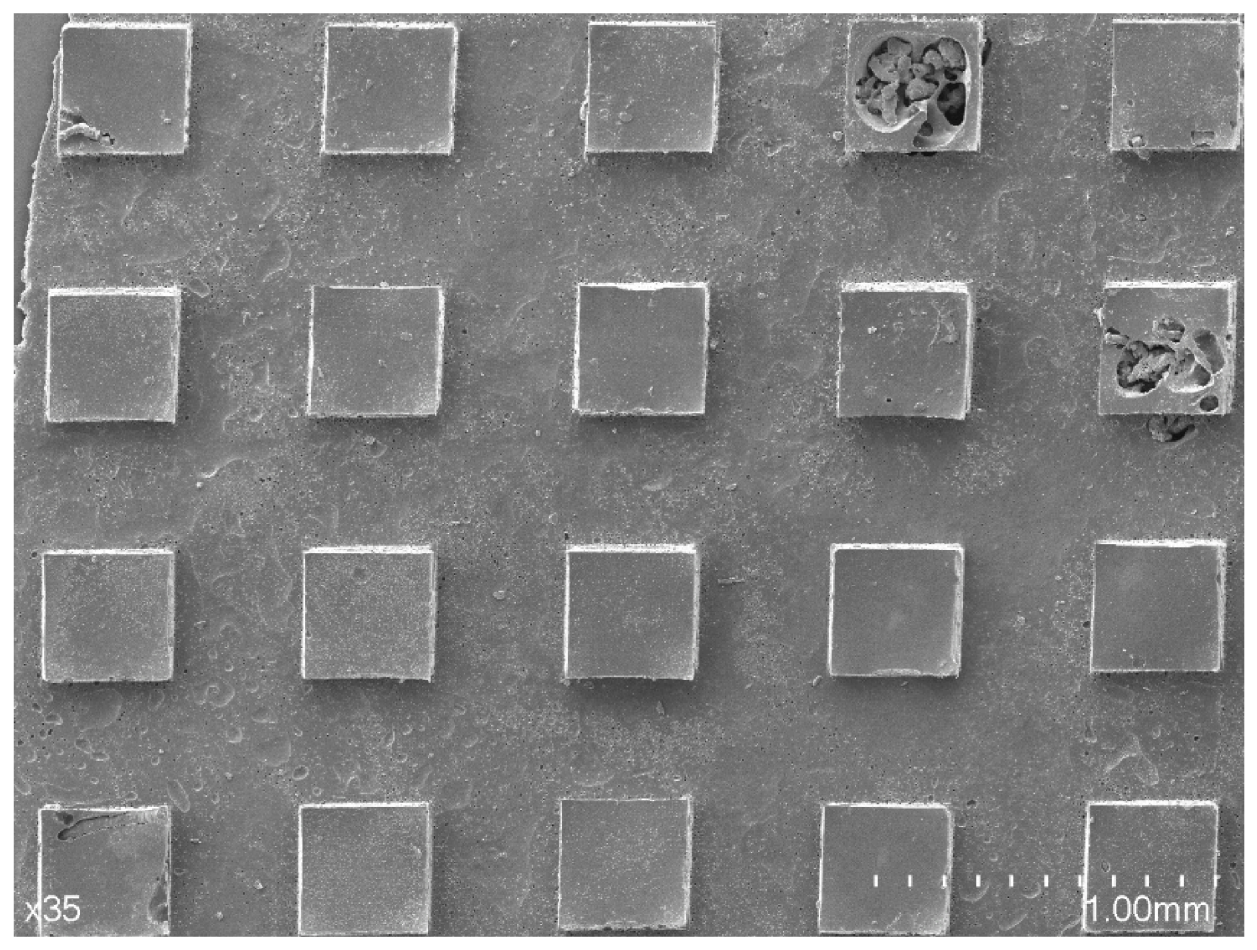
SEM morphology characterization of sensor dielectric layer.

The characterization results of field emission scanning electron microscopy (FE-SEM) are presented in Figure 3, which intuitively verifies the molding performance of the resin template replication process. The morphological images reveal that the microcolumns on the surface of the ionic film are arranged in a highly regular manner with uniform row and column spacing; individual columns remain upright without inclination, with only a small number of fractures generated during peeling, and the side walls of the columns are smooth and intact. Fifty microcolumns were randomly selected for dimensional statistics. The side length of the microcolumns fluctuates within the range of 400 ± 20 μm, the overall dimensional error is controlled within 7%, and the morphological consistency of samples across different batches is excellent. The SEM images of the dielectric layer clearly display the three-dimensional structure of the micro-pillars. The micro-pillars grow perpendicularly on the surface of the film substrate without delamination or peeling between the pillars and the substrate. Continuous and interconnected air buffer layers are formed in the gaps between pillars, which provide sufficient deformation space for graded compression under low pressure. In contrast to the random concave-convex microstructures fabricated from frosted glass, where the protrusions feature irregular heights and local sharp bumps prone to irreversible plastic deformation under compression, resulting in permanent damage to the micro-morphology after repeated compression, the ordered micro-pillars bear uniform force with stress distributed across the entire pillar body without localized stress concentration. No obvious damage occurs to the microstructures after repeated compression and rebound, offering structural support for stable long-term cycling of the device over 20,000 cycles. After applying the maximum full-scale pressure of 1038 kPa, the micro-pillars fully collapse and the gaps close. Re-observation after complete pressure unloading and a 30 s static waiting period shows that the height of micro-pillars and array gaps are basically consistent with those of the original uncompressed samples, with no permanent compaction or channel fracture. This directly proves that the TPU micro-pillar framework possesses fully elastic and reversible rebound properties and elucidates the intrinsic mechanism underlying the device’s low hysteresis and high cycling stability from a microscopic perspective.

## 4. Sensor Performance Test and Comparative Analysis

### 4.1. Comparison of Sensitivity of Dielectric Layers with Different Microstructures

**Figure 4:**
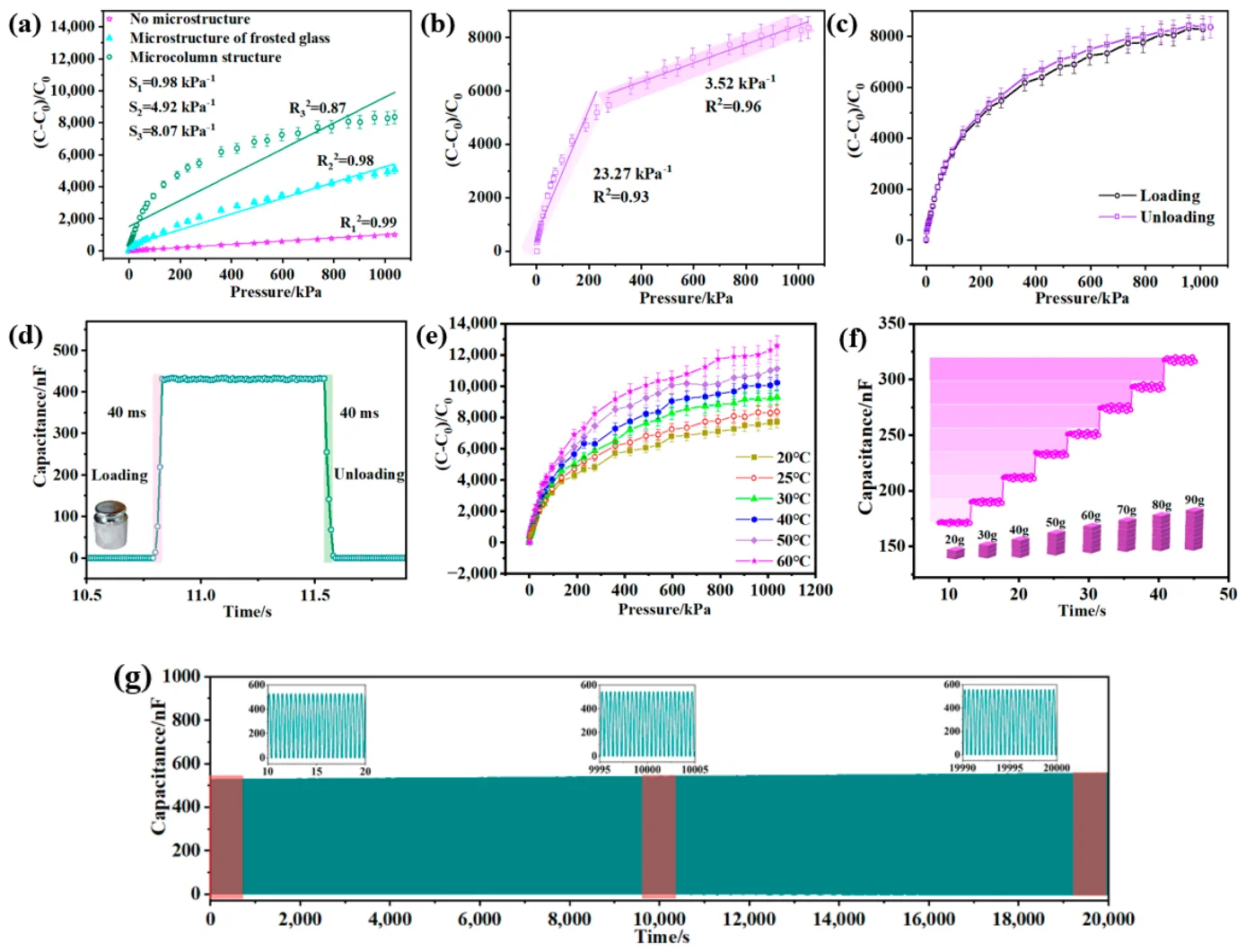
Sensor performance test: (**a**) performance curves of sensors with different microstructures; (**b**) segmented performance curve of the micropillar-structured sensor; (**c**) hysteresis performance test curve of the micropillar-structured sensor; (**d**) loading-unloading response time test curve of the micropillar-structured sensor; (**e**) performance curves of the micropillar-structured sensor at different temperatures; (**f**) linear incremental response of the sensor under linearly increasing pressure and (**g**) performance stability curve of the sensor under more than 20,000 repeated loading cycles.

Synchronous tests were conducted on three types of sensors, namely dense thin films without microstructures, frosted random concave-convex microstructures, and ordered array micropillars, to obtain full-range pressure-normalized capacitance curves ranging from 0 to 1038 kPa, with sensitivities calculated segmentally; the results are presented in Figure 4a. The dense ionic thin-film sensor without microstructures exhibited no segmented response characteristics across the entire pressure range, with an overall average sensitivity of merely 0.98 kPa^−1^. Within the ultra-low pressure range of 0–1 kPa, the variation in normalized capacitance was less than 500, resulting in an extremely low signal-to-noise ratio under weak pressure loads, which hindered the identification of tiny mechanical signals such as pulses and micro-airflows. Although its linear fitting coefficient reached 0.99, the capacitance increased excessively gently with rising pressure, leading to obvious deficiencies in overall sensing performance. This phenomenon is mainly attributed to the absence of air buffer structures inside the dense thin film. Capacitance response is only achieved through slight overall deformation of the thin film under compression, without dynamic expansion of the electrode contact area, which restricts the effective enhancement of both geometric capacitance and electric double-layer capacitance, and eliminates the dual signal amplification mechanism.

The random concave-convex microstructure sensor fabricated using a frosted glass mold can improve the device’s response performance to a certain extent, delivering a full-range average sensitivity of 4.92 kPa^−1^ and enhanced low-pressure detection capacity compared with dense thin films. Nevertheless, the sensitivity varies drastically between low and high pressure regions, with a linear fitting coefficient of 0.98. The disordered distribution and uneven stress bearing of surface concave-convex structures cause irreversible plastic deformation of local protrusions during compression, which readily induces baseline drift after long-term testing and fails to meet the requirements of high-precision quantitative pressure detection. In contrast, the ordered array micropillar ionic thin-film sensor fabricated in this work demonstrates outstanding segmented linear sensing performance. Its response curve can be distinctly divided into two stable linear regions, as illustrated in Figure 4b. In the low-pressure range of 0–200 kPa, the sensitivity reaches 23.27 kPa^−1^ with a linear fitting coefficient of 0.93. The rapid compressive deformation of air layers within micropillar gaps under low pressure drastically enlarges the effective contact area between electrodes and dielectric layers, endowing the device with exceptional capacity for weak pressure recognition, which enables precise detection of low-load signals including pulse fluctuations, trace airflows, and subtle facial expressions. In the medium-to-high-pressure range of 200–1038 kPa, the device maintains a steady sensitivity of 3.52 kPa^−1^ and a linear fitting coefficient of 0.96. Once the micropillar structures are fully compacted, uniform deformation of the elastic TPU substrate delivers stable and continuous signal output, making the sensor suitable for medium- and high-pressure mechanical scenarios such as joint bending, mechanical clamping, and bearing human body weight.

The three groups of comparative test results fully verify that the hierarchically compressible structure of ordered array micropillars simultaneously realizes high-sensitivity response under low pressure and stable linear output over a wide measurement range. Its comprehensive sensing performance is remarkably superior to dense structure-free thin films and random concave-convex microstructure thin films, effectively resolving the longstanding trade-off between sensitivity and detection range in conventional microstructure sensors.

### 4.2. Analysis of Loading-Unloading Hysteresis Characteristics

Three complete cyclic loading-unloading tests ranging from 0 to 1038 kPa were performed on the array micropillar sensor, with the compression and decompression curves recorded synchronously as illustrated in Figure 4c. The results reveal that the averaged compression and decompression curves exhibit high overlap, with a maximum full-range hysteresis error of merely 2.6%. The hysteresis originates from the following two primary sources: first, a slight temporal lag in ion migration within the ionic thin film; second, minor internal friction arising from the elastic rebound of the polymer matrix. The array micropillars disperse stress to reduce the overall deformation resistance of the thin film. Meanwhile, the air layers between micropillar gaps buffer elastic lag and significantly mitigate hysteresis effects. The outstanding resilience of TPU material enables rapid recovery of micropillars after complete pressure removal. No obvious offset is observed in curves from repeated loading-unloading cycles, leading to enhanced accuracy of quantitative pressure detection.

### 4.3. Dynamic Response and Recovery Speed Test

Dynamic response speed serves as a core indicator to evaluate a sensor’s capacity to capture transient pressure signals in real time. In the experiment, a 150 g weight was instantly placed on and swiftly removed from the sensor to simulate dynamic impact loads, while an impedance analyzer collected capacitance variation curves in real time, as presented in Figure 4d. Data show that after the step pressure is applied, it takes 40 ms for the capacitance signal to rise from the initial baseline to 90% of the steady-state peak value; after pressure unloading, it takes 40 ms for the capacitance signal to fall from the peak to 10% of the baseline amplitude. Therefore, both the response time and recovery time of the sensor are 40 ms. The millisecond-level rapid response and recovery performance stems from the structural advantages of array micropillars. The split columnar structure undergoes independent deformation without extensive molecular chain dragging and internal friction in a solid dense thin film. The air layers in gaps generate no viscous resistance during compression and release. The multi-point contact interfaces of micropillars shorten ion migration distances, allowing rapid enrichment and diffusion of anions and cations, which delivers excellent tracking performance for dynamic signals. This sensor can meet the signal acquisition demands of dynamic scenarios including real-time pulse fluctuation monitoring, rapid limb movement tracking and instantaneous airflow impact detection.

### 4.4. Experiment on the Influence of Ambient Temperature on Sensing Performance

To investigate the interference pattern of ambient temperature variations on output signals of the array micropillar iontronic pressure sensor modified by oxygen plasma in practical applications, full-range pressure calibration tests from 0 to 1000 kPa were carried out under constant temperatures of 20 °C, 25 °C, 30 °C, 40 °C, 50 °C and 60 °C respectively. The normalized capacitance-pressure response curves are displayed in Figure 4e. The experimental results indicate that the sensor’s capacitance response shifts upward overall with rising ambient temperature, and the normalized capacitance value (C − C_0_)/C_0_ increases under identical pressure at higher temperatures. The underlying mechanism is as follows: elevated temperatures accelerate the migration rate of anions and cations inside the ionic gel, enlarging the interfacial electric double-layer capacitance. Meanwhile, the elastic modulus of the TPU gel matrix slightly declines under heat, resulting in increased compressive deformation of micropillars under equal pressure, further expanding the effective contact area of electrodes and synchronously boosting geometric capacitance. Within the temperature range of 20–60 °C, the device’s curves maintain highly consistent variation trends, all featuring segmented characteristics of rapid growth under low pressure and decelerated growth under high pressure. No failures in sensitivity, signal distortion or uncontrolled baseline drift are detected. Minor discrepancies exist between curves within the daily human wearable temperature range (25–37 °C), indicating weak temperature interference. For high-temperature industrial applications, algorithm-based temperature compensation can eliminate output offset, verifying the sensor’s wide temperature adaptation window. Quantitative comparative experiments with humidity coupling were carried out to quantitatively evaluate the effect of ambient humidity on the performance of pressure sensors. Using the controlled variable method, stepwise pressure loading tests ranging from 0 to 1000 kPa were performed on the sensor under typical working conditions with humidity varying from 30% RH to 90% RH. The test results are presented in Table 1, which indicate that the sensor possesses excellent resistance to humidity interference. Within the humidity range of 30% RH to 90% RH, the maximum reduction in sensitivity is merely 3.6%. This favorable performance is attributed to the hermetic packaging of the sensor. Accordingly, the influence induced by humidity can be neglected under conventional operating conditions, and a humidity compensation strategy is only required for ultra-high precision detection scenarios.

### 4.5. Static Response Experiment with Stepwise Gradual Weight Loading

Standard 10 g weights were adopted as stepwise constant loads, which were stacked sequentially on the sensor surface from light to heavy mass. Each load level was maintained for 5 s with real-time capacitance values recorded, and the time-capacitance step response curve is shown in Figure 4f. The experimental phenomenon shows that capacitance instantly jumps to a stable plateau after each additional weight, and the steady-state capacitance plateaus corresponding to each load level are independent without overlapping intervals. This characteristic confirms the sensor’s outstanding static stability and its capability to precisely distinguish tiny gradient loads. It is applicable to low-load scenarios such as minor deformation of human joints and faint pulse pressure and can stably identify continuously increasing graded external forces. It provides reliable support for load monitoring in rehabilitation training, graded weight detection of objects and quantitative measurement of static pressure.

### 4.6. Long-Term Cyclic Durability and Stability Test

To verify the long-term continuous operational reliability of the device, a durability test consisting of 20,000 reciprocating loading-unloading cycles within a force range of 0–2 N was carried out. The peak capacitance variation throughout the entire test was fully recorded, and partial waveforms captured at the early and late stages of the cycles were compared, as illustrated in Figure 4g. The test results reveal that the peak output capacitance exhibited no obvious continuous attenuation during the 20,000 cycles, with highly consistent waveform shapes and peak magnitudes. The signal drift of the peak value at the end of the cycles relative to the initial peak was merely 5.1%, and no signal distortion was observed. To investigate the effects of long-term storage and repeated bending on sensor performance, experiments including 60 days of storage at room temperature and more than 500 repeated bending operations were conducted. The experimental results demonstrate that long-term storage and repeated bending exert negligible adverse impacts on the sensor. The excellent durability is attributed to the following two mechanisms: first, the ordered micro-pillars distribute external pressure loads uniformly, preventing permanent plastic deformation of the polymer matrix induced by localized stress concentration; no irreversible damage occurs to the morphology of the micro-pillars after repeated compression. Second, edge sealing with PDMS blocks moisture and dust penetration, preventing ion loss inside the ionic film and interfacial delamination or debonding between electrodes and the dielectric layer, thereby maintaining stable interfacial impedance over long-term cyclic operation. Such durable performance satisfies the application requirements of wearable devices for round-the-clock physiological monitoring over several consecutive weeks and industrial equipment for long-term online pressure detection.

### 4.7. Pressure Sensor Detection Limit Test

**Figure 5:**
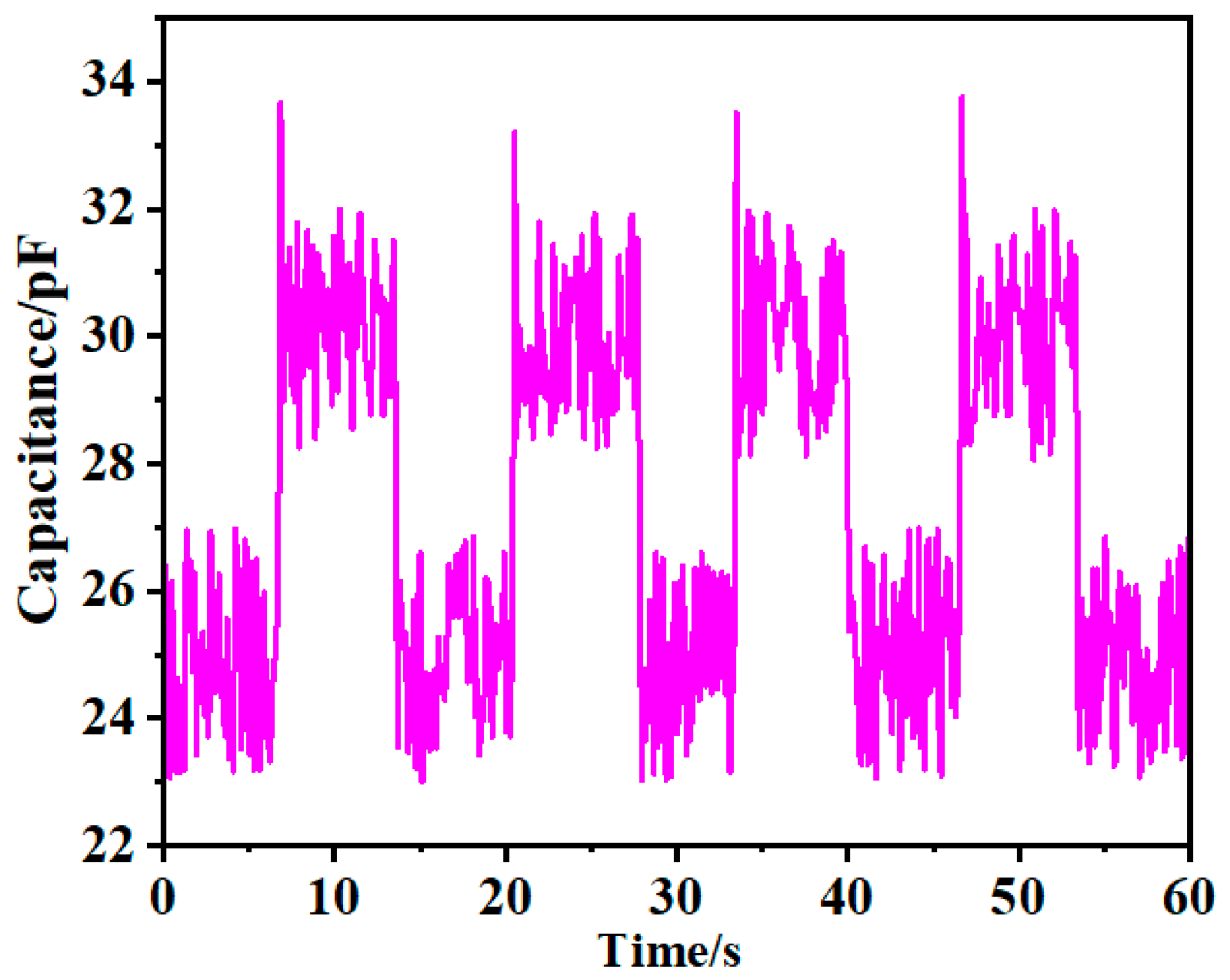
Test for the lower detection limit of the pressure sensor.

To explore the upper and lower detection limits of the pressure sensor, graded pressure loads ranging from 0 to 1200 kPa were continuously applied to the pressure sensor, and the normalized capacitance output signals were recorded simultaneously. The test results indicate that after the external pressure rises to 1038 kPa, the capacitance value of the device no longer increases significantly with rising pressure and the signal becomes saturated. Accordingly, the upper detection limit of this sensor is determined to be 1038 kPa. Restricted by the minimum loading force threshold of the pressure testing machine, the equipment cannot directly apply ultra-tiny loads at the Pascal level. Hence, lightweight sponge counterweights were adopted for the lower limit detection experiment: an ultra-thin sponge sheet weighing merely 0.01 g was selected as the micro-pressure source, loading and unloading operations were repeatedly implemented on the sensing area, and dynamic capacitance output curves were collected in real time (Figure 5). The experimental waveforms are distinct and recognizable; a stable and distinguishable step signal of capacitance is generated when the sponge is loaded, and the baseline fully recovers after unloading. Converted into pressure values, the minimum detectable pressure of the sensor is approximately 1 Pa. Such an ultra-low detection limit enables it to accurately capture subtle physiological pressure signals of the human body including arterial pulse beats, tiny skin deformations and faint airflow impacts, making it highly suitable for low-load application scenarios such as non-invasive home health monitoring, flexible electronic skins and subtle tactile sensing.

### 4.8. Horizontal Comparison with Advanced Offline Flexible Pressure Sensors Developed in Recent Years

Table 2 compares the performance of the sensor designed in this paper with those reported in the existing literature. Experimental tests and research demonstrate that benefiting from the micropillar ionic gel dielectric layer, the proposed sensor possesses remarkable advantages in terms of sensitivity and detection range.

## 5. Wearable Monitoring Experiment of Physiological Signals from Multiple Human Body Parts

Relying on the device’s high sensitivity, fast dynamic response, excellent flexibility and wide measurement range, wearable monitoring application experiments of human physiological signals are carried out to fully verify the practical application value of the device. The sensors are attached to different body surface positions of the human body with medical breathable adhesive tape, causing no obvious foreign body sensation. The sensors can bend synchronously with the skin and joints without signal disconnection or baseline drift.

**Figure 6:**
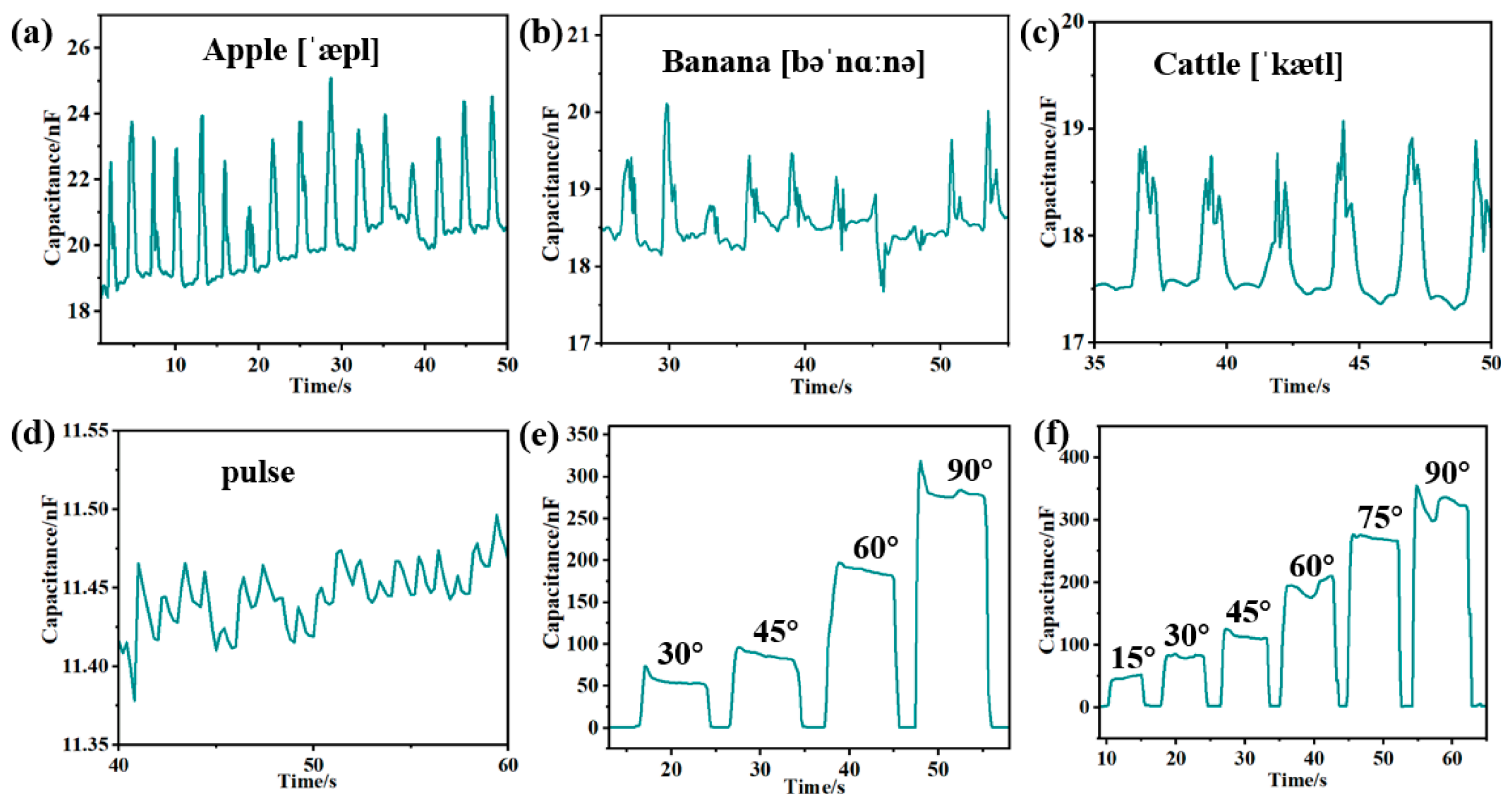
Wearable testing of physiological signals from multiple human body parts: (**a**) capacitance response curve corresponding to the pronunciation of the English word “apple”; (**b**) capacitance response curve corresponding to the pronunciation of the English word “banana”; (**c**) capacitance response curve corresponding to the pronunciation of the English word “cattle”; (**d**) real-time capacitance waveform for human wrist pulse monitoring; (**e**) step capacitance response of gradient bending at the second knuckle of the index finger and (**f**) step capacitance response of gradient flexion and extension of the elbow joint.

1. Laryngeal Voice Recognition: As shown in Figure 6a–c, the sensor is attached to the epidermal layer of the throat for monosyllabic pronunciation tests. When reading single words such as “apple”, “banana” and “cattle” respectively, the vibration of the vocal cords transmits to the larynx to generate differentiated characteristic capacitance curves. The peak value, duration and fluctuation frequency of waveforms corresponding to different sentences have unique identification features, which can assist the rehabilitation training of aphasic patients and the development of flexible sensing equipment for voice recognition.
2. Radial Artery Pulse Monitoring: As shown in Figure 6d, the sensor is attached to the radial artery on the inner side of the wrist to collect pulse waveforms under static conditions. The baseline of the output curve is stable, and the primary pulse peak and secondary wave double characteristic peaks can be clearly distinguished. The waveforms repeat stably in a periodic manner, enabling accurate capture of the number of pulse beats per minute. This technology is applicable to non-invasive home heart rate monitoring and basic cardiovascular screening. The weak pulse pressure is only at the hectopascal level, and the high sensitivity of the device at 23.27 kPa^−1^ ensures a high signal-to-noise ratio free of clutter interference.
3. Multi-angle Motion Monitoring of Finger Joints and Elbows: As shown in Figure 6e,f, the sensor is fastened to the second joint of the index finger (elbow joint) to perform bending movements at various angles in sequence. As the bending angle increases, the pressure borne by the sensor rises synchronously, and the normalized capacitance value increases steadily step by step. The signal intervals corresponding to different angles do not overlap, allowing quantitative identification of joint bending amplitude. It can be applied to quantitative recording of limb movement during post-operative rehabilitation training and tactile feedback systems for intelligent prosthetics.

## 6. Conclusions

In this paper, ionic films are fabricated by compounding imidazolium ionic liquids with a TPU elastic matrix. An ordered array micro-pillar dielectric layer is constructed via a low-cost and recyclable resin template spin-coating and mold-reversing process. A sandwich-structured ionic flexible pressure sensor is assembled by pairing the dielectric layer with inkjet-printed silver flexible PI electrodes. Through systematic regulation of material ratios, micro-morphology characterization, multi-dimensional electrical performance tests and diversified practical application verifications, this work fully elaborates the synergistically enhanced sensing mechanism combining geometric capacitance and interfacial electric double-layer capacitance induced by the array micro-pillar structure. The sensor features an ultra-broad pressure detection range of 0–1038 kPa and exhibits typical piecewise linear response characteristics. Its sensitivity reaches 23.27 kPa^−1^ (R^2^ = 0.93) in the low-pressure range of 0–200 kPa and 3.52 kPa^−1^ (R^2^ = 0.96) in the medium-to-high-pressure range of 200–1038 kPa, enabling simultaneous acquisition of weak physiological signals and detection of conventional mechanical pressure. The device delivers outstanding dynamic sensing performance with both response and recovery times of only 40 ms, a low hysteresis error of 2.6% during loading-unloading cycles, and merely 5.1% attenuation in peak capacitance output after 20,000 repeated loading-unloading durability tests, which endows it with rapid signal feedback, low hysteresis and excellent long-term mechanical and electrical stability. Benefiting from stable and superior comprehensive sensing performance, the device can accurately capture various physiological activity signals including human pulse fluctuations, vocal vibrations, and multi-gradient bending of fingers and elbow joints, demonstrating promising application prospects in wearable health monitoring, quantitative evaluation of postoperative limb rehabilitation, flexible human–machine interaction and other fields. Meanwhile, the entire device fabrication process eliminates the need for expensive micro-nano processing equipment such as photolithography and 3D printing. The adopted raw materials are highly versatile, the micro-structured templates are recyclable, and large-area coating and large-scale production can be realized, which effectively reduces the overall mass production cost and provides a complete and feasible technical implementation route for the industrial and engineering application of high-performance ionic flexible pressure sensors.

## Figures and Tables

**Table 1:** Effect of humidity on pressure sensors.

Relative Humidity/% RH	Zero-Pressure Baseline/pF	0~200 kPa Sensitivity/kPa^−1^	200~1000 kPa Sensitivity/kPa^−1^	Lag Error/%
30	25	23.51	3.52	2.6
40	25	23.27	3.52	2.6
50	26	23.02	3.52	2.6
60	27	22.96	3.51	2.6
70	27	22.83	3.51	2.7
80	29	22.66	3.50	2.9
90	31	22.65	3.49	3.2

**Table 2:** Performance comparison of different types of microstructure sensors.

Microstructure Type	Sensitivity/kPa^−1^	Detection Range/kPa	Response Time/ms	References
bionic lotus leaf microstructure	0.815	0–50	38	[32]
pyramid microstructure	0.55	0–1300	—	[33]
porous grid microstructure	0.09	0–2000	80	[34]
wheat awn-like layered structure	3.12	0–107	13	[35]
microcolumn structure	23.27/3.52	0–1038	40	—

## Data Availability

The data presented in this study are available in this article.
